# Bile Acid Deconjugation and Anti‐*Helicobacter pylori* Activity of *Limosilactobacillus reuteri* DSM 34531

**DOI:** 10.1002/mnfr.70514

**Published:** 2026-07-10

**Authors:** Nize Otaru, Julia K. Bird, Sara Soldi, Vera Atanasova, Valeria Sagheddu, Emiel Ver Loren van Themaat, Jan van Leeuwen, Robert E. Steinert

**Affiliations:** ^1^ Health, Nutrition & Care (HNC) DSM‐Firmenich Kaiseraugst Switzerland; ^2^ Bird Scientific Writing Wassenaar Netherlands; ^3^ AAT‐Advanced Analytical Technologies Fiorenzuola d'Arda, Piacenza Italy; ^4^ Data Science DSM‐Firmenich Delft Netherlands; ^5^ Adelaide Medical School Faculty of Health and Medical Sciences CRE in Translating Nutritional Science to Good Health The University of Adelaide Adelaide Australia

**Keywords:** bile salt hydrolase, co‐aggregation, *Lactobacillaceae*, probiotic, urease

## Abstract

*Limosilactobacillus reuteri* DSM 34531 has been attributed with probiotic characteristics, including a reduction of symptoms of acute diarrhea and atopic dermatitis. Yet, other probiotic features typical for some *Lm. reuteri* strains such as bile salt hydrolase (BSH) and anti‐ Helicobacter pylori activity have hitherto not been documented. Whole genome sequencing of *Lm. reuteri* DSM 34531 was performed to identify BSH genes, followed by BSH classification into previously described BSH clades. Suspended cell pellets and supernatants were tested for BSH activity via a quantitative colorimetric method. Auto‐ and co‐aggregation, and urease activity of single and mixed *Lm. reuteri* DSM 34531 and *H. pylori* DSM 21031T cultures were evaluated using optical density‐ and microscopy‐based approaches. Genome analysis of *Lm. reuteri* DSM 34531 revealed a single BSH‐encoding gene, with its protein sequence clustering closest to the BSH‐T3 phylotype. Corresponding in vitro BSH activity was confirmed. *Lm. reuteri* DSM 34531 co‐aggregated with *H. pylori* DSM 21031T and reduced its urease activity. Conclusively, our data suggest that *Lm. reuteri* DSM 34531 possesses an active BSH, suggesting potential relevance to cholesterol metabolism. We also demonstrate in vitro co‐aggregation with *H. pylori* while reducing its urease activity indicating potential anti–*H. pylori* activity under in vitro conditions.

## Graphical abstract

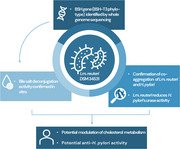

**Abbreviations**
BLAST: Basic Local Alignment Search Tool
BSH: Bile salt hydrolase
GCA: Glycocholic acid
GDCA: Glycodeoxycholic acid
TCA: Taurocholic acid
TDCA: Taurodeoxycholic acid

## 1 Introduction

*Limosilactobacillus reuteri* strains are known for their various beneficial effects and are among the most researched and commercially utilized bacterial strains [1]. While certain core characteristics are commonly observed across several taxonomic groups, such as supporting a healthy gut microbiome or digestive tract, many probiotic effects at the intestinal or extraintestinal level are indeed strain‐specific [2].

Some *Lm. reuteri* strains have been researched with respect to bile salt hydrolase (BSH) activity as it may confer a survival advantage in the gut [3, 4, 5]. In addition, BSH activity may modulate the gut microbiome to prevent dysbiosis, or promote a healthy immune response and enhance barrier function [6]. However, most notable is the hypothetical lowering effect on plasma cholesterol: BSH activity may increase the excretion of deconjugated bile acids, which trigger the liver to synthesize more bile salts de novo from cholesterol. In addition, deconjugated bile salts are less effective at emulsifying fat in the intestine, thereby potentially lowering the absorption of dietary lipids, including cholesterol [7]. Proof of concept has been demonstrated in a human study using a probiotic yoghurt containing a BSH‐producing *Lm. reuteri* strain (NCIMB 30242), which lowered cholesterol in hypercholesterolemic subjects [8]. However, the occurrence of BSH genes in *Lm. reuteri* are strain‐specific, which warrants investigation at the strain level [9].

Certain *Lm. reuteri* strains have also been demonstrated to co‐aggregate with pathogens like *Helicobacter pylori* to enhance their clearance [10, 11]. *H. pylori* is a risk factor for gastric and systemic diseases, and its complete eradication through antibiotic therapy can significantly reduce associated health risks [10]. However, therapeutic failure has increased in recent years due to antibiotic resistance. Moreover, antibiotic treatments often cause side effects such as diarrhea, nausea, and vomiting [10]. Probiotics, including *Lm. reuteri* strains, therefore, represent a promising alternative or adjunctive treatment option. They are hypothesized to show anti‐*H. pylori* activity through several mechanisms, including (i) the production of antimicrobial compounds; (ii) the promotion of mucin synthesis in the intestinal epithelium; (iii) immune modulation; (iv) the inhibition of *H. pylori*’s urease activity; (v) and the prevention of colonization through co‐aggregation [10, 12]. Yet, some characteristics are strain‐specific, indicating the necessity of careful validation prior to making related health claims [11, 13, 14, 15].

*Lm. reuteri* DSM 34531, formerly known as *Lm. reuteri* DSM 12246‐CU was identified in the late 1990s from a panel of 47 selected lactobacilli that were tested in vitro for their probiotic potential. *Lm. reuteri* DSM 34531 demonstrated excellent pH tolerance and adhesion properties as well as strong antimicrobial activity [16, 17]. In follow‐up human clinical trials, its persistence in the gastrointestinal tract was confirmed [16, 17, 18] and host health benefits were documented in combination with *Lacticaseibacillus rhamnosus* 19070–2 or as part of multistrain mixtures. This included clinical studies demonstrating a reduction of acute diarrhea symptoms and atopic dermatitis in young children, as well as a reduction in crying and fussing in infants [19, 20, 21, 22, 23]. To date, there is no data available on BSH activity or anti‐*H. pylori* properties. Therefore, in this series of in vitro experiments, we sought to investigate the BSH activity of *Lm. reuteri* DSM 34531, as well as its co‐aggregation ability and effect on the urease activity of *H. pylori*.

## 2 Experimental Section

### 2.1 Bile Salt Hydrolase Activity: In Silico and In Vitro Analyses

**FIGURE 1:**
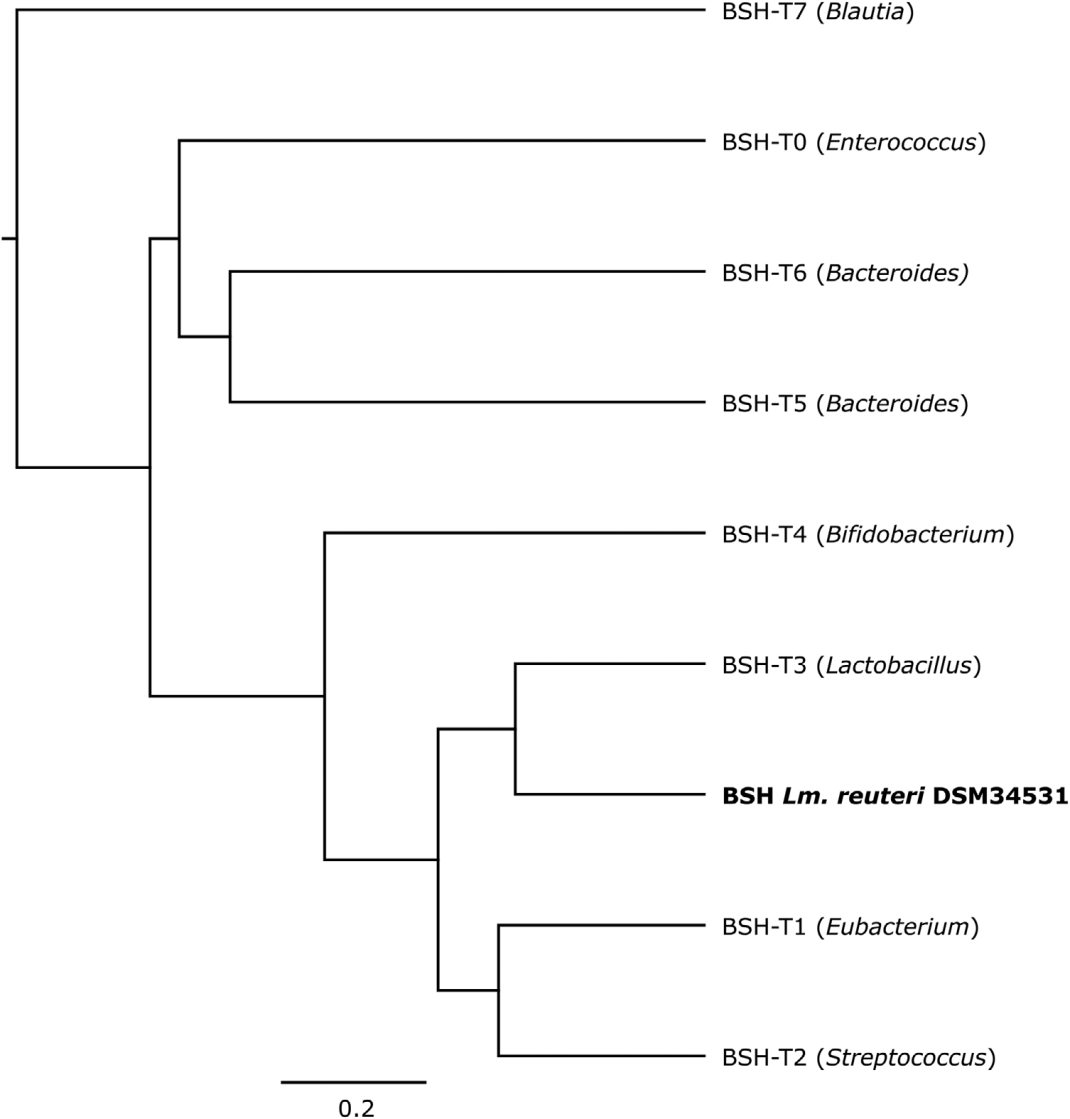
Phylogenetic tree based on the BSH protein sequence comparing the *Lm. reuteri* DSM 34531 BSH sequence to reference sequences of eight previously described BSH phylotypes reported by Song et al. [25].

A single vial of *Lm. reuteri* DSM 34531was sent to BaseClear B.V. (Leiden, the Netherlands) from the DSM‐Firmenich internal strain conservation unit. Sample identity was confirmed by Sanger sequencing of the 16S rRNA gene, which identified the strain as *Lm. reuteri* with 99.76% sequence identity using the validated MicroSeq database. To identify BSH coding genes, the genome of *Lm. reuteri* DSM 34531 was sequenced by BaseClear B.V. using a combination of Illumina NovaSeq 6000, producing paired‐end 150 nt sequences from a nextera XT library, and PacBio Sequel, producing approximately 5000 nt long sequences. The genome assembly was made using ABySS (v2.0.2) and scaffolded using a combination of BLASR (v1.2.1) and SSPACE‐LongRead (v1.0). GapFiller (v1.1) was used to fill remaining gaps, and pilon (v1.21) to correct nucleotide mismatches between reads and scaffolds. The genome was annotated using PROKKA [24]. The sequencing reads were assembled into a genome of 2.039 Mb in 9 scaffolds with 1978 predicted genes. No virulence factors or antibiotic resistance genes were identified using the Comprehensive Antibiotic Resistance Database (CARD v4.0.1) and the Virulence Factors of Pathogenic Bacteria database (VFDB; downloaded on May 4, 2026). Predicted genes were aligned against both databases using the Basic Local Alignment Search Tool (BLAST) with sequence identity > 80% and target coverage > 70% as thresholds. The PacBio read coverage of the genome was 153X, and Illumina coverage was 1954X. To identify genes encoding for BSHs, the BLAST with two reference BSHs sequences, BSH1 from *Lactiplantibacillus plantarum* (Uniprot ID: B9V401) and BSH2 from *Lp. plantarum* (Uniprot ID: B9V3Z8), was used. BLAST hits with an E‐value <0.05 were regarded as being significant. Phylogenetic analyses of the protein sequence were performed based on Clustal Omega multiple alignment for Figure 1 or pairwise global alignment for Supporting Information Figure S1 using Jukes–Cantor as distance and UPGMA for Figure 1 or neighbor‐joining for Supporting Information Figure S1 clustering to build the phylogenetic tree with 100 bootstraps as implemented in Geneious 2022.1.1. A lactobacilli reference group previously described by O'Flaherty et al. [9] was used for Supporting Information Figure S1, comprising 26 diverse lactobacilli BSH sequences. Three sequences (*Lb. johnsonii* 100 100 (AF297873.1), *Lp. plantarum* (S51638.1), and *Lg. salivarius* (JX120368.1)) were not included due to unavailability at the time of analysis (July 14, 2025). Representatives of eight previously described BSH phylotypes from human gut microbiome strains [25] were used as comparators for Figure 1. Analysis of the longest sequence identity was performed according to the method of Needleman and Wunsch [26]. A biodiversity search for homologous BSH sequences was performed using the BLAST, querying the UniProt database and including BSH phylotypes reference sequences [25]. Structural modelling and bile acid substrate docking were done using Boltz‐2 [27].

BSH activity was also determined in vitro. *Lm. reuteri* NCIMB 30242, previously described as exhibiting BSH activity and showing cholesterol‐lowering effects in humans [28], served as a positive control. *Lm. reuteri* NCIMB 30242 and *Lm. reuteri* DSM 34531 were grown on MRS agar plates (Becton, Dickinson and Company, Franklin Lakes, NJ, USA) for 72 h at 37°C under anaerobic conditions. Following verification of colony purity and viability, a single colony from each strain was cultured in MRS broth under microaerophilic conditions. For each culture, 2 mL was centrifuged at 16249 *g* for 10 min. The supernatant was collected, and cell pellets were washed twice with sterile demineralized water. A BSH activity assay was performed according to Allain et al. [29] and Grill et al. [30], with minor modifications. In short, 100 µL supernatants or washed cells were mixed with 2.4 g/L of conjugated bile salts (glycocholic acid (GCA), glycodeoxycholic acid (GDCA), taurocholic acid (TCA), and taurodeoxycholic acid (TDCA) (Merck KGaA, Darmstadt, Germany)) and incubated at 37°C for 30 min. The reaction was terminated by adding 15% trichloroacetic acid, and the solution was centrifuged to precipitate proteins. Supernatants were then mixed with 680 µL of 0.3 M borate buffer with 1% sodium dodecyl sulfate (pH 9.5), and 80 µL of 0.3% picrylsulfonic acid solution, and incubated for 30 min at room temperature in the dark. Subsequently, 0.6 mM HCl was added to stop the reaction. Each condition was assessed using two independent replicates. The amount of glycine or taurine released was measured via optical density at 416 nm (OD_416_) and a standard curve with free glycine or taurine (from 0 to 14 mM).

### 2.2 Anti‐*H. pylori* Activity: Auto‑ and Co‑Aggregation Assays

Auto‐ and co‐aggregation were evaluated using optical density‐ and microscopy‐based approaches. In a first step, *Lm. reuteri DSM* 34531 (obtained from DSM‐Firmenich) and *H. pylori* DSM 21031T (obtained from the German Collection of Microorganisms and Cell Cultures (DSMZ)) were incubated overnight at 37°C. *Lm. reuteri* was grown in MRS medium while *H. pylori* was grown in Brucella broth (Liofilchem, Roseto degli Abruzzi, Italy) supplemented with 5% fetal bovine serum (EuroClone, Pero, Italy). Cultures were centrifuged for 20 min at 3000 *g*, washed twice in PBS, and resuspended in simulated gastric juice (sodium taurocholate (0.08 mM), phospholipids (0.02 mM), sodium chloride (34 mM), hydrochloric acid (59 mM)) at pH 3.4 ± 0.2 to mimic the environment of the postprandial stomach. The optical density at 600 nm (OD_600_) of bacterial suspensions was adjusted to 0.25 ± 0.05 (A_0_), which corresponds to a cell concentration of approximately 2 × 10^8^ CFU/mL. Suspensions were vortexed and incubated for 1 h (A_1h_) and 2 h (A_2h_) at 37°C for auto‐ and co‐aggregation assays to correspond with gastric emptying rates under physiological conditions. For co‐aggregation assays, cell suspensions were mixed at a ratio of 1:1 (*H. pylori* DSM 21031T: *Lm. reuteri* DSM 34531). Each condition was assessed using three independent replicates. Quantification of auto‐ and co‐aggregation was performed as described by Zawistowska‐Rojek et al. [31]. In short, the auto‐aggregation percentage was calculated as specified in Equation (1).

(1) $$\text{Auto-aggregation}\;\left[\%\right]=1-\frac{A_{t}}{A_{0}}\times 100$$

*A*_0_ represents the OD_600_ at *t* = 0, and *A*_t_ represents the OD_600_ at subsequent time points. Co‐aggregation percentage was calculated as specified in Equation (2).

(2) $$\text{Co-aggregation}\;\left[\%\right]=\left(\frac{\left(A_{x}+A_{y}\right)/2-A_{x+y}}{\left(A_{x}+A_{y}\right)/2}\right)\times 100$$

*A*_x_ and *A*_y_ represent the OD_600_ of the two strains incubated separately, while *A*_x+y_ represents the OD_600_ of the mixture at each time point.

In a second step, agglomerate formation at 2 h was assessed using optical microscopy. Bacterial suspensions were Gram‐stained to allow for the differentiation of *H. pylori* 21031T (Gram‐negative) and *Lm. reuteri* DSM 34531 (Gram‐positive).

### 2.3 Anti‐*H. pylori* Activity: Urease Activity Assay

Given that urease is central to the metabolism and virulence of *H. pylori* [32], a urease assay was performed on single and mixed cultures to assess the urease activity of *H. pylori* 21031T in the presence and absence of *Lm. reuteri* DSM 34531 using a modified phenol red method as described by Zhao et al. [33]. In short, 500 µL urease reaction buffer (20% urea and 0.012% phenol red, dissolved in phosphate buffer, pH 6.5) was added to 500 µL of pure or mixed culture suspensions in test cuvettes from auto‐ and co‐aggregation assays. The cuvettes were incubated for 1 h at 37°C, and optical density at 562 nm (OD_562_) was measured to quantify the formation of pink color due to pH change induced by ammonia formation during urea hydrolysis. Thus, the formation of pink color indicates the presence of active urease. Each condition was assessed using three independent replicates.

### 2.4 Data Analyses and Visualization

Statistical analyses and visualizations were performed using GraphPad Prism (v 10.2.2). Group comparisons were made using the Welch's *t‐*test, with pairwise comparisons by group where appropriate. Adjustment for multiple comparisons was performed using the Benjamini–Hochberg method. For the bile acid deconjugation data, a linear regression with interpolation of the data was calculated using GraphPad Prism (v 10.2.2). Significance level was set to *p* ≤ 0.05.

## 3 Results

### 3.1 *Lm. Reuteri* DSM 34531 Possesses an Active Bile Acid Hydrolase

BSH presence in *Lm. reuteri* DSM 34531 was investigated in silico. Using two reference sequences from *Lp. plantarum* (BSH1 and BSH2) as queries, a single putative BSH coding gene (Prokka ID 00924) was identified in the *Lm. reuteri* DSM 34531 genome. The BSH protein encoded by this gene exhibited 74.5% sequence identity with *Lp. plantarum* BSH1 (97.4% sequence coverage) and 70.0% sequence identity with *Lp. plantarum* BSH2 (89.9% sequence coverage). The identified gene and protein sequences are deposited at GenBank NCBI under the GenBank accession number PV387302.

**FIGURE 2:**
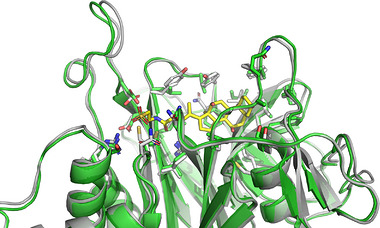
Close‐up Boltz2 model of the *Lm. reuteri* DSM 34531 BSH (green) with TCA bound (yellow) and a Boltz2 model of the representative BSH for clade BSH‐T3 (gray).

Phylogenetic analysis of the BSH protein sequence, using a lactobacilli reference group previously described by O'Flaherty et al. [9], revealed high similarity with a BSH from another *Lm. reuteri* strain 100–23 (Supporting Information Figure S1), confirming the species taxonomy and indicating close evolutionary relatedness to other BSH‐specific strains. Further phylogenetic analysis of the protein sequence, compared to reference sequences representing eight previously described BSH phylotypes from human gut microbiome strains [25], revealed that the DSM 34531 BSH sequence clusters within the BSH‐T3 phylotype (Figure 1). This classification was further supported by analysis of the longest sequence identity (according to the method of Needleman and Wunsch), which showed the highest identity (60%) with the BSH‐T3 representative and lower identities (29%–53%) with representatives of the other clades. Key amino acid positions (25) involved in catalysis or substrate binding were identified in the *Lm. reuteri* DSM 34531 sequence (2; 16; 18–20; 24; 56; 64; 65; 77–79; 98; 99; 129; 133–136; 138; 171; 172; 221; 224 and 270). All catalytic amino acids and half of the amino acid positions for substrate binding were conserved in the closest BSH hit sequences. This suggests that the 325 amino acid‐long protein is catalytically active, but it also confirms the occurrence of high diversity within the BSH family. A biodiversity search using BLAST, including the BSH phylotypes reference sequences and additional open‐access data (UniProt), revealed that the BSH‐T3 clade reference sequence had 72% identity with the DSM 34531 BSH. No hits were found among representatives of other clades (Supporting Information Table S1). At least ten of the 25 key amino acid positions displayed high variability, suggesting altered catalytic properties within the BSH family (Supporting Information Figure S2). In total, 29 hits with 100% identity, calculated over the selected 25 key positions, were identified, including strains of *Lm. reuteri* and *Limosilactobacillus albertensis* (Supporting Information Table S1). Furthermore, the BSH of *Lm. reuteri* DSM 34531 exhibited a similar three‐dimensional structure with the representative of clade BSH‐T3 (Figure 2).

**FIGURE 3:**
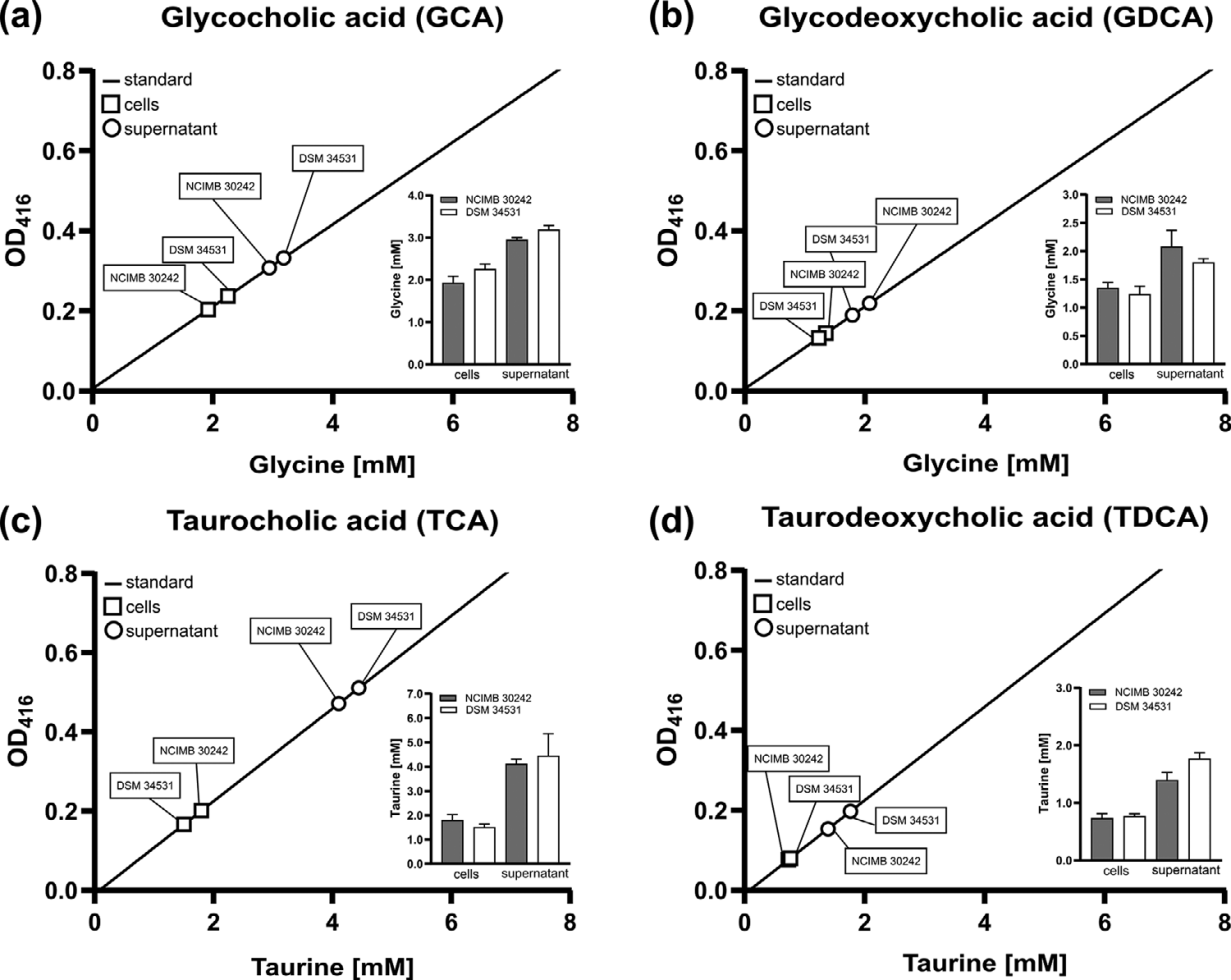
Bile salt hydrolase activity of *Lm. reuteri* DSM 34531 and positive control *Lm. reuteri* NCIMB 30242, displayed as released free glycine and taurine from conjugated bile salts. The main panel depicts linear regression of free glycine or taurine, with interpolated mean values for both strains. The insert bar plot displays the mean released glycine and taurine of two independent replicates. Error bars represent the standard deviation. (a) Glycine is released from glycocholic acid. (b) Glycine is released from glycodeoxycholic acid. (c) Taurine is released from taurocholic acid. (d) Taurine is released from taurodeoxycholic acid.

Subsequently, BSH activity was determined in vitro using washed cells and supernatants. Overall, supernatants showed higher BSH activity than washed cells (Figure 3a–d) and TCA and GCA showed to be better substrates than TDCA and GDCA. OD_416_ measurements further suggested that the BSH activity of *Lm. reuteri* DSM 34531 is comparable to that of the positive control *Lm. reuteri* NCIMB 30242, with no significant differences between the two strains (Figure 3a–d).

### 3.2 *Lm. Reuteri* DSM 34531 Co‐Aggregates With *H. pylori*

**FIGURE 4:**
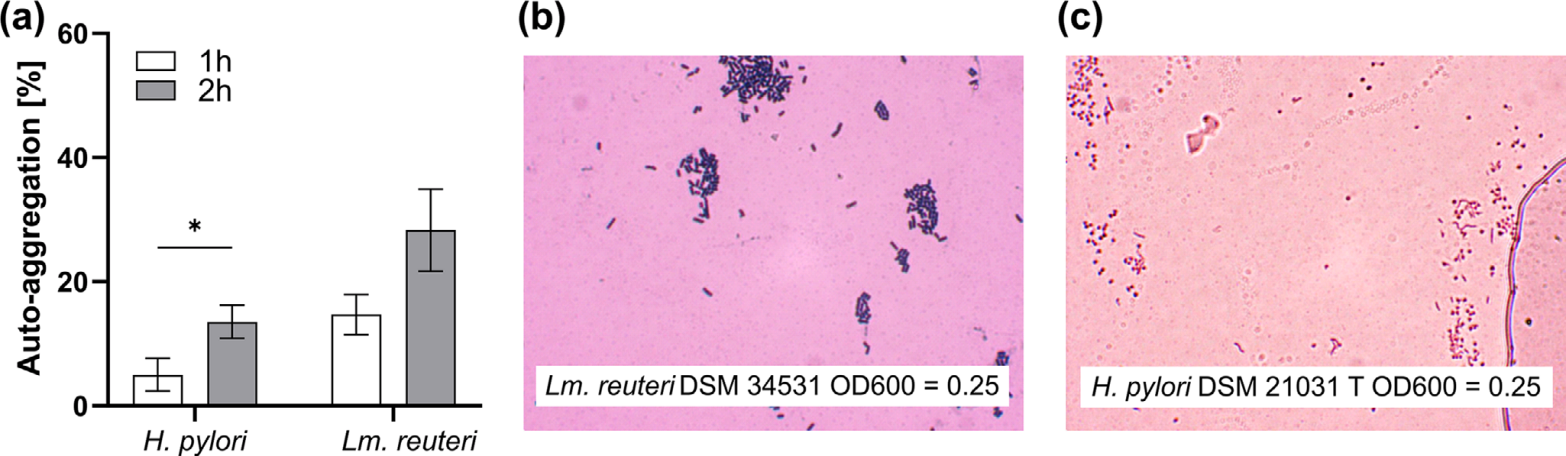
Auto‐aggregation of *Lm. reuteri* DSM 34531 and *H. pylori* DSM 21031T using cells at an OD_600_ of ∼0.25 for both strains, incubated for 1 and 2 h in simulated gastric juice. (a) Mean percentage of auto‐aggregation for three independent replicates of both strains at 1 and 2 h incubation. Error bars represent the standard deviation; *p < 0.05 (b) Optical microscopy of *Lm. reuteri* DSM 34531 at 100x magnification at 2 h incubation. (c) Optical microscopy of *H. pylori* DSM 21031T at 100x magnification at 2 h incubation.

Based on OD_600_ measurements, both *H. pylori* and *Lm. reuteri* showed a time‐dependent increase in auto‐aggregation activity, which was significant for *H. pylori* (*p* < 0.05) (Figure 4a). In addition, auto‐aggregation was confirmed microscopically based on the formation of individual strain agglomerates at 2 h (Figure 4b,c).

**FIGURE 5:**
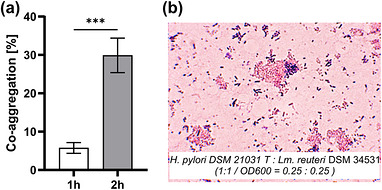
Co‐aggregation of *Lm. reuteri* DSM 34531 and *H. pylori* DSM 21031T (1:1) using cells at an OD_600_ of ∼0.25 for both strains, incubated for 1 and 2 h in simulated gastric juice. (a) Mean percentage of co‐aggregation for three independent replicates of both strains at 1 and 2 h incubation. Error bars represent the standard deviation; *** *p* < 0.001 (b) Optical microscopy of cell suspension at 100x magnification at 2 h incubation.

There was also a time‐dependent increase in co‐aggregation of *H. pylori* and *Lm. reuteri* based on OD_600_ measurements, at 1 and 2 h (*p* < 0.001; Figure 5a). Moreover, co‐aggregation was confirmed microscopically based on the formation of mixed strain agglomerates at 2 h (Figure 5b).

### 3.3 *Lm. Reuteri* DSM 34531 Decreases Urease Activity of *H. pylori*

**FIGURE 6:**
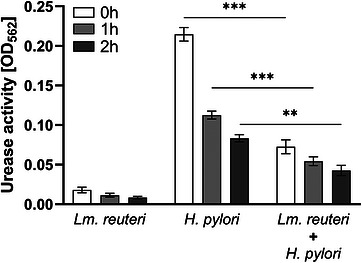
Mean urease activity for three independent replicates of *Lm. reuteri* DSM 34531, *H. pylori* DSM 21031T, and a mixture of both strains (1:1), incubated for 0 h, 1 and 2 h in simulated gastric juice. Urease activity is expressed as optical density at 562 nm (OD_562_). Error bars represent the standard deviation; ** *p* (FDR‐adjusted) < 0.01; *** *p* (FDR‐adjusted) < 0.001.

Urease activity of *H. pylori* was high and steadily decreased during the 2 h incubation, in contrast to *Lm. reuteri* DSM 34531, which showed low urease activity. When cell suspensions of *H. pylori* and *Lm. reuteri* were mixed, urease activity was significantly (*p* < 0.001, FDR‐adjusted) lower than in the *H. pylori* single culture suspension at 0 h and further declined during the 2 h incubation (Figure 6).

## 4 Discussion

*Lm. reuteri* DSM 34531, formerly known as *Lm. reuteri* DSM 12246‐CU has been shown to possess various probiotic health benefits in humans, including a reduction of symptoms of acute diarrhea and atopic dermatitis [19, 20, 21, 22, 23]. Here, we report additional in silico and in vitro evidence showing that *Lm. reuteri* DSM 34531 possesses an active BSH. The in silico screening revealed that *Lm. reuteri* DSM 34531 possesses a single BSH gene with no homologs, which is in line with observations for other *Lm. reuteri* strains [9]. Protein sequence analysis classified the BSH as a member of the BSH‐T3 clade, previously described as one of three clades comprising lactobacilli based on phylogeny and functional characterization [25]. Lactobacilli of the BSH‐T3 clade exhibit the highest in vitro deconjugation activity when compared to those from other clades [34]. While some protein features of BSHs are conserved, such as the N‐terminal cysteine nucleophile in the mature protein, other protein features, including the binding pocket, such as the substrate‐binding loops, are variable [35]. Here we show that at least 10 out of the 25 key amino acid positions display high variability amongst the identified 254 BLAST hits, confirming the occurrence of high diversity within the BSH family [36]. We further show that the key amino acids for substrate binding 100% match with 29 lactobacilli BSHs available in open access reference banks, including *Lm. reuteri* CRL 1098, which has previously been shown to reduce cholesterol in hypercholesterolemic subjects upon four weeks of daily intake in probiotic yoghurt [37]. In line with this, we also found in vitro that *Lm. reuteri* DSM 34531 exhibits BSH activity comparable to that of *Lm. reuteri* NCIMB 30242, another strain with a well‐characterized BSH that has been associated with clinically meaningful cholesterol reductions in hypercholesterolemic subjects after six to nine weeks of daily supplementation [8, 28]. Microbial BSH activity has been linked to host cholesterol homeostasis via the modulation of the secondary bile acid pool. BSH enzymes enhance the excretion of bile acids via deconjugation, thereby limiting their reabsorption during enterohepatic circulation, which promotes de novo hepatic formation of primary bile acids from cholesterol [7]. In line with this mode of action, another *Lm. reuteri* strain belonging to the BSH‐T3 clade was previously shown to modulate enterohepatic bile acid concentrations in mice [34]. In addition, deconjugated bile acids are less effective than conjugated bile acids in emulsifying lipids, which is believed to reduce dietary cholesterol absorption [7].

We further provide new in vitro evidence showing that *Lm. reuteri* DSM 34531 co‐aggregates with *H. pylori* and reduces its urease activity under physiologically relevant conditions mimicking the environment of the postprandial stomach. Notably, a decrease in urease activity was already detectable at 0 h incubation in the urease assay, likely due to prior incubation during aggregation tests. The release of urease in the stomach contributes to *H. pylori* virulence by catalyzing the breakdown of urea into ammonia, raising the pH of the stomach and enabling *H. pylori* to proliferate [12]. Several modes of action have been proposed for the urase inhibition activity of lactobacilli strains, including (i) production of antimicrobial compounds thereby limiting activity of *H. pylori* [38], and (ii) a direct downregulation of the urease gene expression, thereby inhibiting synthesis of urease [39]. Indeed, *Lm. reuteri* has long been known to produce the antimicrobial molecules reuterin, acetate, and lactate [38]. Limiting urease activity may also be beneficial further down in the gastrointestinal tract, where microbial urease expression has been linked to increased ammonia production in the colon with detrimental effects on host health in susceptible individuals [40]. On the other hand, the observed co‐aggregation of *Lm. reuteri* DSM 34531with *H. pylori* may limit the pathogen's mobility, and adhesion to the gastric mucosa and promote its excretion [11]. Taken together, these in vitro data suggest that *Lm. reuteri* DSM 34531 may contribute to improved clearance of *H. pylori* during medical treatment. Indeed, another probiotic strain with similar in vitro characteristics, namely *Lm. reuteri* DSM 17648 (Pylopass^TM^) is well established to contribute to *H. pylori* eradication and improvement of related gastrointestinal symptoms [11, 41, 42].

A number of experimental limitations warrant consideration. With respect to the effects on *H.pylori*, it should be noted that the OD‐based auto‐ and co‐aggregation assay is semi‐quantitative, only allowing for relative comparisons rather than absolute measurements. Also, while the microscopy is suggestive of co‐aggregation between *Lm. reuteri* and *H. pylori*, the Gram stain procedure can disturb cell arrangement and introduce artifacts [43]. Finally, these experiments were performed in vitro and therefore cannot fully capture the heterogeneous conditions to which probiotics are exposed in real‐world use. Consequently, neither similar behavior of the strain in the human gastrointestinal tract nor a measurable clinical effect can be directly inferred from these in vitro findings. Human studies aimed at demonstrating clinical benefits and elucidating the mechanism of action would validate these novel probiotic activities of *Lm. reuteri* DSM 34531.

In conclusion, *Lm. reuteri* DSM 34531 possesses an active BSH, as demonstrated by in silico and in vitro analyses indicating its potential to lower cholesterol in hypercholesterolemic subjects. In addition, *Lm. reuteri* DSM 34531 co‐aggregates with *H. pylori* and reduces *H. pylori* urease activity, potentially aiding in its clearance from the body. Human clinical studies are warranted to further validate these observations.

## Author Contributions

**R.E.S**., **V.A**.: Conceptualization, **V.S**., **E.V.L.v.T** and **J.v.L**.: methodology, **J.K.B**., **V.S**., **E.V.L.v.T** and **J.v.L**.: formal analysis, **S.S**. and **V.S**.: investigation, **N.O**., **J.K.B**., and **R:E:S**.: writing – original draft preparation, **N.O**., **J.K.B**., **S.S**., **V.S**., **E.V.L.v.T**, **J.v.L**., and **R.E.S**.: writing – review and editing, **N.O**., **J.v.L**.: visualization, **R.E.S**. and **S.S**.: supervision. All authors have read and agreed to the published version of the manuscript.

## Funding

This study was funded by DSM‐Firmenich.

## Conflicts of Interest

N.O., R.E.S., E.V.L.v.T., V.A., and J.v.L. are employees of DSM‐Firmenich. V.S. and S.S. are employees of Advanced Analytical Technologies that received funding from DSM‐Firmenich to perform this research. J.K.B. is an employee of Bird Scientific Writing who received funding from DSM‐Firmenich to contribute to the writing of this manuscript.

## Supporting information




**Supporting File 1**: mnfr70514‐sup‐0001‐Figures.docx.

**Supporting File 2**: mnfr70514‐sup‐0002‐TableS1.xlsx.

## Data Availability

The data that support the findings of this study are included in the article and Supporting Information. The gene and protein sequences are openly available in GenBank at https://www.ncbi.nlm.nih.gov/genbank/, reference number PV387302. Further inquiries can be directed to the corresponding author.
