# Supplementary material for: Bile Acid Deconjugation and Anti‐Helicobacter pylori Activity of Limosilactobacillus reuteri DSM 34531
Source: Mol Nutr Food Res. 2026 Jul 10;70(13):e70514. doi: 10.1002/mnfr.70514 (PMC13352470; doi:10.1002/mnfr.70514)
Supplement: Supplementary file 1 — Supporting File 1: mnfr70514‐sup‐0001‐Figures.docx. [file MNFR-70-e70514-s002.docx]

**Supporting Information Figures**


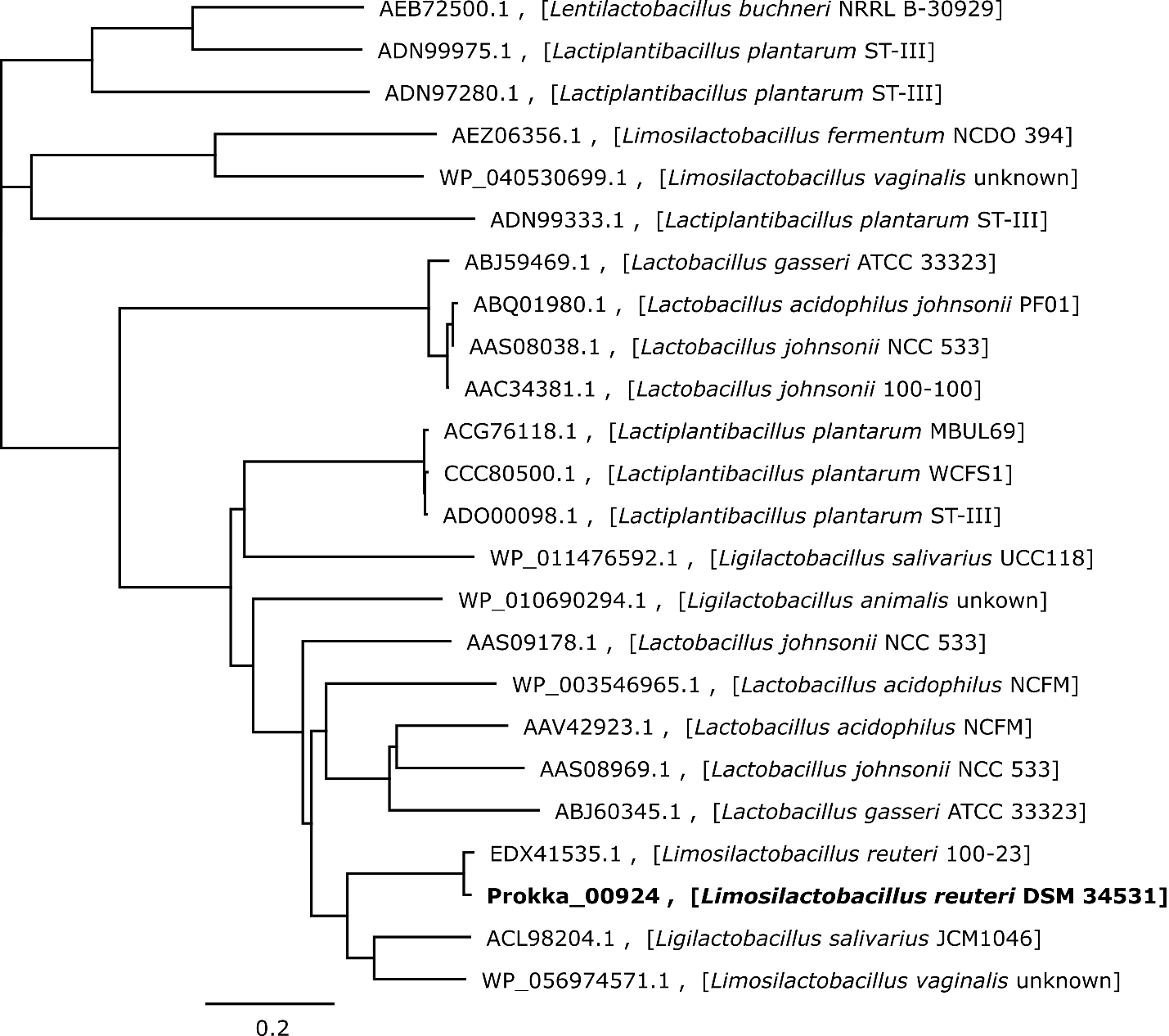


**Supporting Information Figure S1: Phylogenetic tree based on the BSH protein sequence comparing the *L. reuteri* DSM 34531 BSH sequences to reference sequences of 23 lactobacilli, previously described by O’Flaherty et al.** ^[9]^**.**


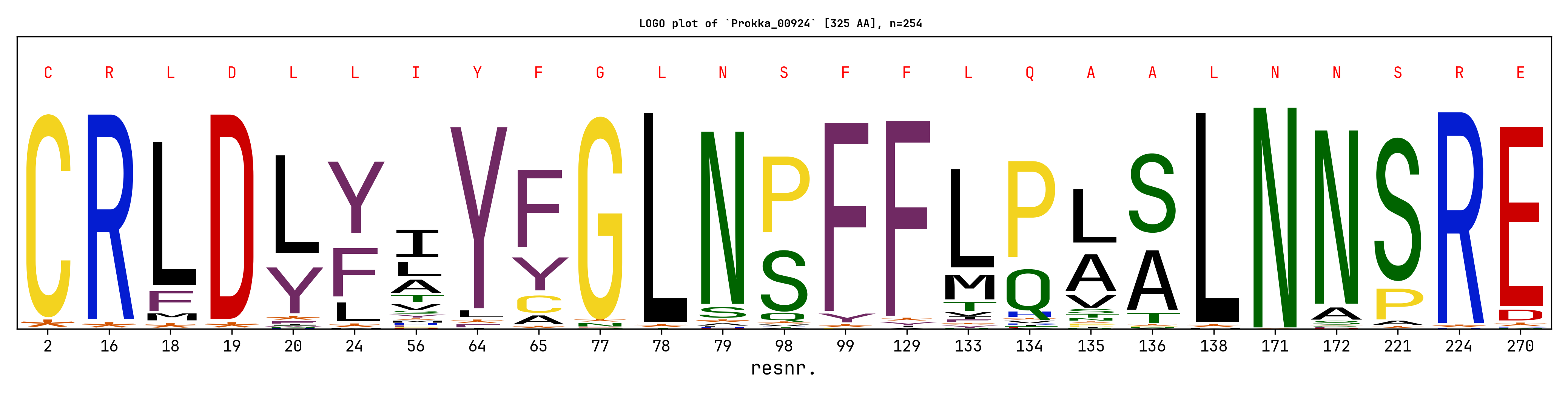


**Supporting Information Figure S2: Sequence logo covering the 25 key amino acid positions calculated over 254 BLAST hits*.***
